# Compounds from *Viburnum sargentii* Koehne and Evaluation of Their Cytotoxic Effects on Human Cancer Cell Lines 

**DOI:** 10.3390/molecules15074599

**Published:** 2010-06-25

**Authors:** Ki-Eun Bae, Han-Soo Chong, Dong-Sup Kim, Young-Woong Choi, Young-Sook Kim, Young-Kyoon Kim

**Affiliations:** Department of Forest Products and Biotechnology, College of Forest Sciences, Kookmin University, Seoul, 136-702, Korea; E-Mail: kieun@kookmin.ac.kr (K.B.)

**Keywords:** *Viburnum sargentii*, Caprifoliaceae, furcatoside A, 9'-*O*-methylvibsanol, lareciresinol, Cancer cells, antitumor activity

## Abstract

Compounds were isolated from a methanol extract of the dried stem barks of *Viburnum sargentii* Koehne. The structures of the compounds, namely 9'-*O*-methylvibsanol (3), furcatoside A (4) and lareciresinol (5) were elucidated by analysis of spectroscopic data and comparison with values for previously known analogues. In addition, (+)-catechin (1), (+)-epicatechin (2) were also isolated. This work also examined the cytotoxic effects of three compounds 3-5 (25-100 µM) in MCF-7 and A549 cells after 24, 48 and 72 h of exposure. Our results showed that 9'-*O*-methylvibsanol (3) exhibited strong concentration-dependent anticancer effects according to the MTT assay and produced morphological changes consistent with apoptosis, as confirmed by Ho3342 staining analysis revealed that more apoptotic cells were observed after 9'-*O*-methylvibsanol (3) treatment.

## 1. Introduction

Cancer is the second leading cause of death worldwide after cardiovascular diseases. Despite many therapeutic advances, mortality is still unacceptably high [1]. Most of the drugs used today in the clinic were first discovered from plants and microorganisms [2], therefore natural products are increasing interest and importance to cancer patients. In addition, these therapeutic agents have been reported to exert their antitumor effects by inducing apoptosis. Apoptosis, or programmed cell death, is an essential event that plays an important role in organism development and homeostasis. Apoptosis is a tightly regulated process characterized by cell shrinkage, plasma membrane blebbing, and chromatin condensation that is consistent with DNA cleavage in ladders [3,4]. Therefore, the induction of apoptotic cell death is an important mechanism in the anticancer properties of many drugs. During the course of screening plant materials possessing cell disordering activities toward cancer cells, the extract of *V. sargentii* Koehne was identified. The *Viburnum* genus (Caprifoliacea) comprises more than 200 species, shrubs or trees, mainly distributed in Southern America and in Asia and very abundant in the Chinese spontaneous flora [5]. *Viburnum* species are commonly used in folk medicine for their diuretic, antispasmodic and sedative properties, mainly for uterine excitability [6]. The genus *Viburnum* is known to be rich in iridoid glycosides, characterized by a sugar moiety at C-11 and an isovaleroyl group at C-1 (Valeriana-type iridoids) that have been isolated from several *Viburnum* species [7,8,9,10,14,21]. In addition, the known phytochemical studies carried out on Caprifoliaceae species have revealed triterpenoids [11], phenolic compounds [12] and benzofuran-type lignans [13]. Phytochemical studies on *V. sargenti* have documented the occurrence of Valeriana-type iridoid glucosides: 7,10,2’-triacetylsuspensolide F, 1, and viburnoside IV and V [14]. 

The detection of marked cytotoxicity effects against several cancer cell lines including A549 (human lung adenocarcinoma), MCF-7 (human breast adenocarcinoma) in the methanolic extract of *V. sargentii* prompted us to further investigate the constituents of this plant. To our knowledge, no report has been issued on the anticancer effects of *V. sargentii* extracts, therefore, this plant was selected for the current study, aimed at the systematic separation, structural elucidation and biological evaluation of the secondary metabolites responsible for the observed activity.

## 2. Results and Discussion

**Figure 1:**
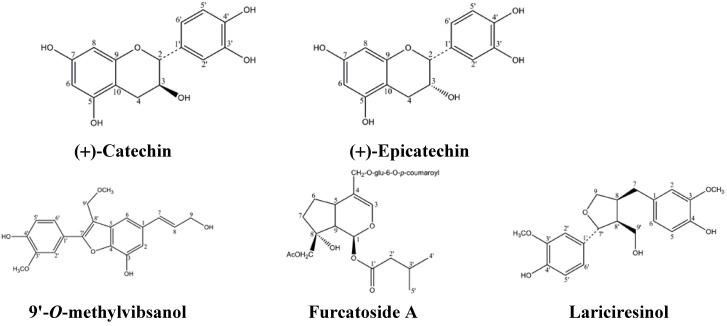
Compound structures from *Viburnum sargentii* Koehne.

The MeOH extract of the stem bark of *V. sargentii* was subjected to solvent-solvent partitioning, to yield hexane, dichloromethane, ethyl acetate and water soluble phases. The ethyl acetate extract exhibited cytotoxic activity on MCF-7, A549 cells. Fractionation of this extract by vacuum liquid chromatography resulted in two main fractions, E_1_~E_2_. The main constituent of active fraction E_1_ was subjected to multiple chromatographic purifications, that afforded compounds 1 and 2 which were proved to be (+)-catechin (1), (+)-ephicatechin (2), respectively, and have already been evaluated and been found to possess antiinflammation, anticancer and diabetes properties. E_2 _fraction was subjected to multiple chromatographic purifications, and afforded compounds 3, 4 and 5 which were proven to be 9'-*O*-methylvibsanol (3), furcatoside A (4) and Lariciresinol (5) through ^1^H, ^13^C and mass spectral data respectively (Figure 1). 9'-*O*-methylvibsanol (3), furcatoside A (4), Lariciresinol (5) had been previously isolated from *Viburnum awabuki* [13], *Viburnum furcatum* [10], *Wikstroemia elliptica* [22] respectively. Lariciresinol (5) were reported to exhibit antioxidant, cytotoxic effects in MCF-7 cells [22,23], but no previous biological studies appear to have been performed on 9'-*O*-methylvibsanol (3).

Compound 3 had the molecular formula C_20_H_20_O_6, _suggesting the addition of an extra methyl groupto vibsanol. The above similarity and difference suggest that 9'-*O*-methylvibsanol (3) should be also a benzofuran-type lignan having an extra methoxy group on C-9 or C-9′ in vibsanol. Methoxy signal at δ_H_ 3.46 showed correlation with an isolated osymethylene (C-9′) at δ_C_ 64.5. Thereby, the methoxy group was placed at the C-9′ position, and the structure of compound 3 was elucidated as 9'-*O*-methylvibsanol (3). 9'-*O*-methylvibsanol (3) had been previously isolated from *Viburnum awabuki* [13]. 

Compound 4 have the 8′, 10′, 11′-oxygen substituted iridoid skeleton with an *iso*-valeroyl group at C-1. The aromatic protons of a *p*-coumaroyl group showed two *trans*-olefinic proton resonances at δ_H_ 6.4 and 7.7 (1H each, *d*, *J* = 15.9 Hz). The spectroscopic evidence led to its identification as furcatoside A that had been previously isolated from *Viburnum furcatum* [10]. Lariciresinol (5), similarly identified by spectroscopic analysis and comparison with literature data, had previously isolated from *Wikstroemia elliptica* [22].

The effect of compounds 3, 4 and 5 on the cytotoxicity of MCF-7, A549 cells was examined first. Cells were treated with various concentrations of compound 3, 4, 5 (25–150 µM) for indicated time periods (24, 48 and 72 h) and cell cytotoxicity was then assessed using the MTT colorimetric assay [15].

**Figure 2:**
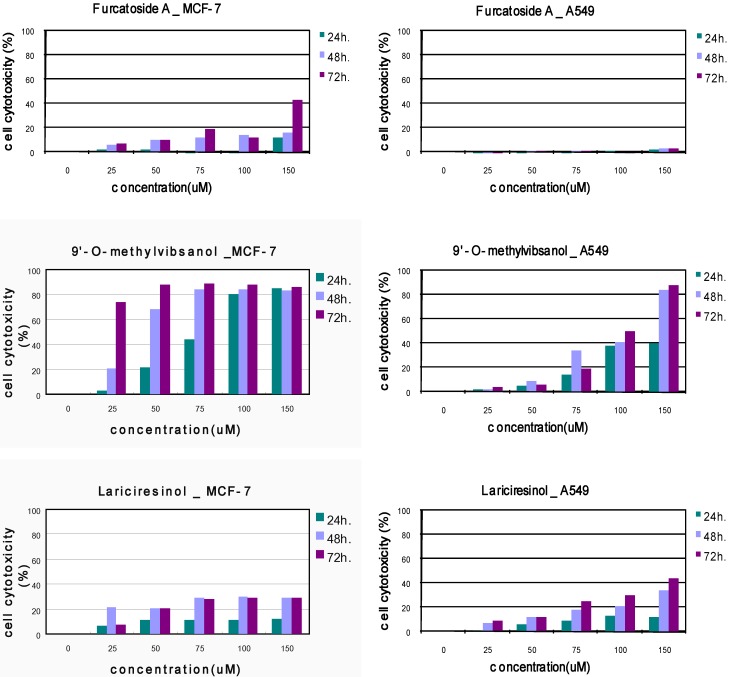
Cytotoxic effects of Compound 3-5 on MCF-7, A549 cell lines.

As shown in Figure 2, treatment of MCF-7, A549 cells with compounds 3-5 resulted in a marked dose-dependent cytotoxicity. The IC_50_ values of compounds 3-5 showed that compound 3 showed the most significant cytotoxicity against MCF-7 cells (Table 1). 

**Figure 3:**
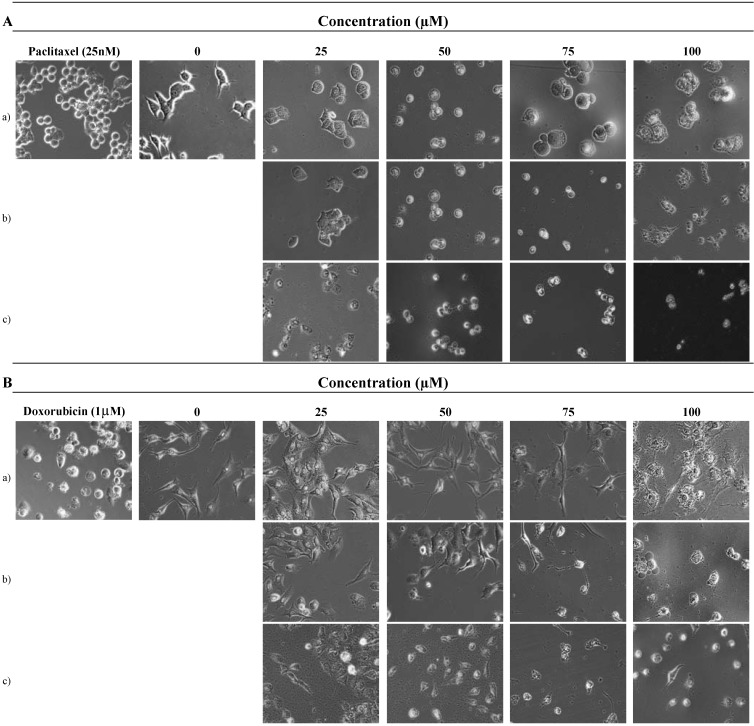
9'-*O*-methylvibsanol (3) induced apoptosis in MCF-7, A549 cells. Morphological change of (A)MCF-7, (B)A549 cells observed under an inverted phase contrast microscope (400 ×).

Since, compound 3 had strong inhibitory effects on MCF-7 and A549 cells growth, the appearance of morphological changes for the cells were observed in the concentration ranges from 25 to 100 µM for 24, 48 and 72 h exposures for further mechanistic studies. Doxorubicin (1 µM) and paclitaxel (25 nM) were used as positive controls. In the next experiment, direct observation using a phase contrast microscope revealed that numerous morphological changes occurred in cells treated with 9'-*O*-methylvibsanol (3). Figure 3 shows these morphological changes of cells after: a) 24 h, b) 48 h and c) 72 h. After incubation with 9'-*O*-methylvibsanol (3), the cellular morphology of (A) MCF-7, (B) A549 cells was severely distorted and cells became round in shape. Also, the cells showed a reduction in cell volume, destabilization of the plasma membrane, indicating an increasing progression toward cell death in a dose-dependent manner. The untreated cells displayed normal, healthy shape with a distinct cytoskeleton.

**Table 1:** Cytotoxic effects of compounds in tumor cell lines. IC_50_ (µM).

substance	MCF-7			A549		
	24 h	48 h	72 h	24 h	48 h	72 h
9'-*O*-methylvibsanol	83.2	43.2	29.3	175.6	104.7	103.0
Furcatoside A	432.9	233.3	180.0			
Lariciresinol	686.1	259.6	281.4	539.1	225.3	168.8
Doxorubicin	39.6	18.3	11.4	16.3	9.7	6.9
Paclitaxel	0.062	0.060	0.055	0.36	0.11	0.09

To elucidate whether 9'-*O*-methylvibsanol (3) inhibits the proliferation of MCF-7, A549 cells by inducing apoptosis, cells treated with compound **3** were examined after Ho33342 staining. The nuclei changes in MCF-7 cells were also observed under a fluorescent microscope (200 ×).

**Figure 4:**
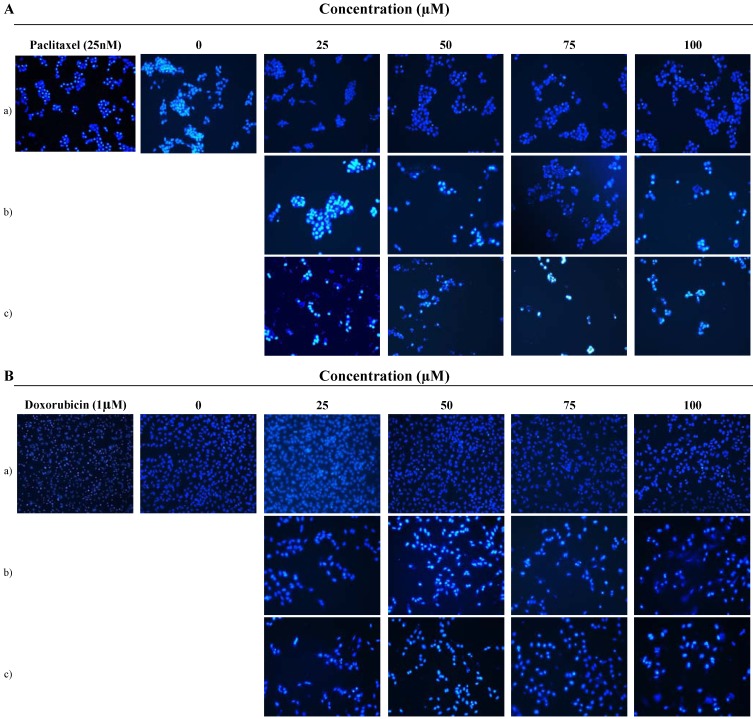
9'-*O*-methylvibsanol (3) treated MCF-7 cells were stained by Ho33342. Nuclei morphologic changes observed by fluorescent microscopy (200 ×). (A) MCF-7, (B) A549 cells, respectively.

As shown in Figure 4, control cells emitted a blue fluorescence with consistent intensity, indicating that the chromatin was equivalently distributed in the nuclei. Following incubation with 9'-*O*-methylvibsanol for a) 24 h, b) 48 h and c) 72 h, the fluorescence light was denser and brighter compared to untreated control cell. Also, the cells showed chromatin condensation and karyopyknosis, which were typical apoptotic phenomena. Also cells showed typical apoptotic phenomena with chromatin condensation and karyopyknosis. Paclitaxel (25 nM) and Doxorubicin (1µM) were used as positive control, (A) MCF-7, (B) A549 cells, respectively.

## 3. Experimental

### 3.1. Extraction

The stem bark of *V. sargentii* was collected in Kyung dong market, Korea, in May 2008. One of the authors identified the plant, and a voucher specimen(YK07081) has been deposited at kookmin university, Korea. Dried stem bark of *V. sargentii* (6.37 kg) was extracted with MeOH (8 L) three times at 46 ºC. The extracts were filtered and evaporated to dryness under vacuum to afford a brown gum (515.15 g) that was partitioned between dichloromethane and water and then the water layer was re-extracted with ethyl acetate. The two organic phases were evaporated to obtain dichloromethane (115.52 g) and ethyl acetate (121.10 g) extracts spots of which were monitored by thin layer chromatography eluting with *n*-hexane-EtOAc system. The spots were visualized by heating silica gel plates sprayed with 15% H_2_SO_4_ in ethanol.

### 3.2. Isolation

The ethyl acetate fraction (41.3 g) was subjected to vacuum column chromatography on silica gel (400 mesh) column, eluting with a gradient of *n*-hexane-EtOAc (5:1~0:1), to yield two subfractions E_1_~E_2_. Subfraction E_1_ (24.7 g) was further separated by normal-phase silica gel (230~300 mesh) column chromatography, eluting with dichloromethane-MeOH (12:1), to yield subfractions E_1_-1~E_1_-2. Subfraction E_1_-2 (1.94 g) was purified by reverse-phase silica gel column chromatography, eluting with MeOH-water (1:5), to afford compound **1** (140 mg) and compound **2** (100 mg). E_2 _(2.57 g) was further separated by normal-phase silica gel column chromatography, eluting with dichloromethane-MeOH (20:1), to afford subfractions E_2_-1~E_2_-5. Subfraction E_2_-1 was separated by normal-phase silica gel column chromatography, eluting with hexane-ethyl acetate (1:2) to yield compound **5 **(10 mg). Subfraction E_2_-3 was further separated by normal-phase silica gel (230~300 mesh) column chromatography, eluting with dichloromethane-MeOH (25:1), to yield compound **3** (20 mg). E_2_-5 was further purified by reverse-phase silica gel column chromatography, eluting with MeOH-water (1:1.2), to isolate compound **4 **(50 mg). The structures of compounds were elucidated on the basis of UNIT-INOVA 300MHz NMR and mass spectral analysis with Micromass ZQ detector (Waters, Milford Massachusetts, USA). The chemical shifts are given in δ (ppm) and coupling constants in Hz.

9'*-O-methylvibsanol ***(3). **C_20_H_20_O_6, _(300MHz, CD_3_OD) 6.87 (1H, d, *J* = 1.5 Hz), 7.16 (1H, d, *J* = 1.2 Hz), 6.63 (1H, d, *J* = 15.6 Hz), 6.30 (1H, d, *J* = 15.6, 6 Hz), 4.24 (2H, d, *J* = 6 Hz), 7.46 (1H, d, *J* = 1.8 Hz), 6.89(1H, d, *J* = 8.4 Hz), 7.32 (1H, dd, *J* = 8.4 Hz, 1.8), 4.62 (2H, s), 3.93 (3H, s), 3.46 (3H, s); 133.5 (C-1), 108.3 (C-2), 142.2 (C-3), 142.2 (C-4), 132.2 (C-5), 108.8 ((C-6), 131.3 (C-7), 127.3 (C-8), 62.7 (C-9), 122.0 (C-1′), 110.5 (C-2′), 147.9 (C-3′), 147.7 (C-4′), 115.3 (C-5′), 120.6 (C-6′), 155.5 (C-7′), 111.2 (C-8′), 64.5 (C-9′), 55.2 (3’-OCH_3_), 57.1 (9'-OCH_3_). ESI-MS (negative ion mode): m/z 649.104 [M-H]^-^. 

*Furcatoside A*** (4). **C_32_H_42_0_14,_ (300MHz CD_3_OD) 6.0 (1H, d, *J* = 6 Hz). 6.62 (1H, bs), 2.01(3H, s), 2.2 (2H, d, *J* = 6.6 Hz), 0.95 (3H, d, *J* = 6.6 Hz), 3.3-4.8 (6H), 6.4/ 7.7 (1H, d, *J* = 15.9 Hz), 7.5 (3H, d, *J* = 8.4 Hz); 91.5 (C-1), 140.7 (C-3), 115.3 (C-4), 36.4 (C-5), 28.7 (C-6), 38.0 (C-7), 80.7 (C-8), 4.67 (C-9), 71.6 (C-10), 70.0 (C-11), 172.8 (COO), 20.8 (COOCH_3_), 173.1 (C-1′), 44.3 (C-2′), 26.8 (C-3′), 22.7 (C-4′), 22.7 (C-5′), 101 .9 (C-1″), 71.3 (C-2″), 75.3 (C-3″), 71.8 (C-4″), 78.1 (C-5″), 62.7 (C-6″), 127.1 (C-1′′′), 131.3 (C-2′′′,6′′′), 116.9 (C-3′′′,5′′′), 161.4 (C-4′′′), 146.8 (C-7′′′), 115.2 (C-8′′′), 168.3 (C-9′′′), ESI-MS (negative ion mode): m/z 355.04 [M-H]^-^. 

*Lariciresinol **(5*****): **C_20_H_24_O_6_, (300MHz CD_3_OD) 2.42 (1H, s), 2.54 (1H, dd, *J* = 13.2 Hz, 10.9), 2.72 (1H, s), 2.95 (1H, dd, *J* = 13.2 Hz, 4.8 Hz), 3.77 (2H, s), 3.88 (3H, s), 3.90 (3H, s), 3.93 (1H, s), 3.99 (1H, dd, *J* = 8.4 Hz, 6.3 Hz), 4.75 (1H, d, *J* = 7.2 Hz), 4.99 (1H, s), 6.62-6.86 (6H, aromatic H); 132.3 (C-1), 112.0 (C-2), 146.5 (C-3), 144.6 (C-4), 114.3 (C-5), 121.0 (C-6), 32.5 (C-7), 42.7 (C-8), 72.3 (C-9), 134.5 (C-1′) 108.6 (C-2′), 146.8 (C-3′), 145.9 (C-4′), 114.0 (C-5′), 118.6 (C-6′), 82.8 (C-7′), 52.5 (C-8′), 59.2 (C-9′), 55.1 (3, 3′-OCH_3_), ESI-MS (negative ion mode): m/z 358.85 [M-H]^-^. 

### 3.3. Cell culture and drug treatment

Human cancer cell line, A549 (human lung adenocarcinoma), MCF-7 (human breast adenocarcinoma), used in this work was purchased from the Korean Cell Line Bank (KCLB). A549, MCF-7 cells were cultured in Dulbecco’s modified eagle medium (DMEM) supplemented with 10% (v/v) fetal bovine serum (FBS), 100 U/ml penicillin, and 100 μg/mL streptomycin, in a humidified incubator containing 5% CO_2_ at 37 ºC. Compounds isolated from *V. sargentii* were dissolved in dimethyl sulfoxide (DMSO) and the final DMSO concentration in all cultures was 0.5%. 

### 3.4. Cell cytotoxicity assay

The 3-(4,5-dimethylthiazol-2-yl)-2,5-diphenyltetrazolium bromide (MTT) assay is a common assay for cell cytotoxicity [15]. Briefly, growing cells were seeded at 1 × 10^5^ cells/well in 96 well plates. After incubation for 24 h. at 37 ºC, cells were exposed to various concentrations of 9'-*O*-methylvibsanol (**3**), furcatoside A (**4**) and Lariciresinol (**5**) and incubated for 12-72 h. Doxorubicin (1–10 µM), Paclitaxel (25 nM–1 µM) were used as positive controls; negative control groups used same amount of DMSO. After incubation, the medium was removed and cells in each well were incubated with PBS contained 5 mg/mL MTT for 4 h. at 37 ºC in 5% CO_2_ incubator. MTT solution was then discarded and 100 μL of DMSO was added into each well to dissolve insoluble formazan crystals. Plates were then kept agitation for 30 min at room temperature for complete solubilization. The level of colored formazan derivative was analysed on a ELISA reader (Opsys MR^TM^, Dynex) at a wavelength of 570 nm. Results were expressed as the mean percentage of cell growth inhibition. The IC_50_ value was expressed as the concentration of compounds that inhibited the growth of cells by 50%. (Figure 2).

### 3.5. Determination of morphological changes of cells

#### 3.5.1. Observation of cells by phase contrast microscope

Cells (5 × 10^4^ cells/well) were incubated for 24 h in 12 well plates. After incubation, the cells were untreated or treated with 9'-*O*-methylvibsanol (3) of four different concentrations for 24–72 h. Then the medium was removed and cells in wells were washed once with PBS. They were observed by phase contrast inverted microscope (Nikon, Japan) at 400 × magnification (Figure 3). Doxorubicin (1 µM) and Paclitaxel (25 nM) were used as positive control for the A549 and MCF-7 cells, respectively. 

#### 3.5.2. Benzimidazole Ho33342 staining

Benzimidazole Ho33342 staining of MCF-7, A549 cells was performed to evaluate the cell death pattern induced by increasing concentrations of 9'-*O*-methylvibsanol (**3**). After 12, 24 and 72 h of incubation, cells were washed with PBS and fixed with methanol for 10 min at room temperature. Fixed cells were washed with PBS, and stained with 1 ug/mL benzimidazole Ho33342 solution for 30 min at incubation. The cells were washed twice more with PBS and analyzed with a fluorescence microscope (Figure 4). Doxorubicin (1 µM) and Paclitaxel (25 nM) were used as positive control, A549 and MCF-7 cells, respectively.

## 4. Conclusions

Recently, a number of studies have demonstrated that apoptosis is of significant importance in the cytotoxic mechanism of chemotherapeutic agents in tumor cells. Apoptosis is a fundamental form of cell death which also plays a role in the development and homeostasis of multicellular organisms. During the course of screening plant materials possessing cell cytotoxic effects toward cancer cell lines, the extract of *V. sargentii* Koehne was selected in this experiment. We have isolated five compounds from stem bark of *V. sargentii*. Among these compounds, 9'-*O*-methylvibsanol **(3)** showed highly cytotoxicity of MCF-7, A549 cells with the lowest IC_50_ range (83.2 to 29.3 µM). Furthermore, the anti-tumor effects 9'-*O*-methylvibsanol **(3)** on MCF-7, A549 cells has not been reported previously. Consequently, we examined whether the apoptotic pathway is involved in the cell death caused by 9'-*O*-methylvibsanol **(3)** in MCF-7, A549 cells. Firstly, we found that the MCF-7, A549 cells treated with 9'-*O*-methylvibsanol **(3)** acquired apoptotic morphological features such as becoming round in shape, reduction in cell volume, destabilization of the plasma membrane, indicating an increasing progression toward cell death in a dose-dependent manner (Figure 3). To elucidate whether 9'-*O*-methylvibsanol **(3)** inhibits the proliferation of MCF-7, A549 cells by inducing apoptosis, cells treated with compound were examined after Ho33342 staining. Results showed that MCF-7, A549 cells treated with 9'-*O*-methylvibsanol **(3)** displayed typical apoptotic phenomena with chromatin condensation and karyopyknosis (Figure 4). These results indicated that compound **3** induces apoptosis of MCF-7, A549 cells. These results may provide a basis for the potential therapeutic application of 9'-*O*-methylvibsanol and its related compounds to cancer therapy. 

However, further studies are needed to determine the mechanism of apoptosis pathway. In general, depending on the cell, apoptosis can be initiated in two ways: by extrinsic pathway or by an intrinsic pathway. In the former, plasma membrane death receptors are involved and the apoptosis signal is provided by interaction between the ligand and the death receptor, which then activates caspase-8 and apoptotic cell death [16,17]. However, intrinsic pathway is triggered by the permeabilization of mitochondrial membranes, furthermore by releasing cytochrome c and ATP levels [18]. Cytochrome c and other apoptotic factors lead to the activation of caspase 9, which finally activates pro-caspase 3 to caspase-3. The activated caspases cleave cellular proteins and via caspase activated DNase (CAD) also chromatin [19,20]. Therefore, in-dept studies are needed to identify the apoptotic pathway of 9'-*O*-methylvibsanol **(3)** for its development as a cancer chemopreventive and/or anticarcinogenic agents.
